# No smile like another: adult age differences in identifying emotions that accompany smiles

**DOI:** 10.3389/fpsyg.2014.00480

**Published:** 2014-05-27

**Authors:** Michaela Riediger, Markus Studtmann, Andrea Westphal, Antje Rauers, Hannelore Weber

**Affiliations:** ^1^Max Planck Research Group “Affect Across the Lifespan,” Max Planck Institute for Human DevelopmentBerlin, Germany; ^2^Institute for Psychology, University of GreifswaldGreifswald, Germany

**Keywords:** smile expressions, age differences, emotion recognition, Duchenne, adulthood

## Abstract

People smile in various emotional contexts, for example, when they are amused or angry or simply being polite. We investigated whether younger and older adults differ in how well they are able to identify the emotional experiences accompanying smile expressions, and whether the age of the smiling person plays a role in this respect. With this aim, we produced 80 video episodes of three types of smile expressions: positive-affect smiles had been spontaneously displayed by target persons as they were watching amusing film clips and cartoons. Negative-affect smiles had been displayed spontaneously by target persons during an interaction in which they were being unfairly accused. Affectively neutral smiles were posed upon request. Differences in the accompanying emotional experiences were validated by target persons' self-reports. These smile videos served as experimental stimuli in two studies with younger and older adult participants. In Study 1, older participants were less likely to attribute positive emotions to smiles, and more likely to assume that a smile was posed. Furthermore, younger participants were more accurate than older adults at identifying emotional experiences accompanying smiles. In Study 2, both younger and older participants attributed positive emotions more frequently to smiles shown by older as compared to younger target persons, but older participants did so less frequently than younger participants. Again, younger participants were more accurate than older participants in identifying emotional experiences accompanying smiles, but this effect was attenuated for older target persons. Older participants could better identify the emotional state accompanying smiles shown by older than by younger target persons. Taken together, these findings indicate that there is an age-related decline in the ability to decipher the emotional meaning of smiles presented without context, which, however, is attenuated when the smiling person is also an older adult.

## Introduction

Facial expressions are a pivotal component of non-verbal communication. They can convey information about the emotional state of the expressive person (e.g., Ekman, 1972; Izard, 1994), and about accompanying behavioral intentions and action requests (e.g., Fridlund, 1994, 1997). Facial displays can thus help to coordinate and regulate social interactions, provided, of course, that the perceiver recognizes the emotional states associated with the expression and responds accordingly (Keltner et al., 2006). This, however, is not always easy because people can show similar facial expressions in disparate emotional situations. A prime example is smiling. Even though smiling is regarded in many cultures as a prototypical sign of pleasure (e.g., Ekman, 1972; Elfenbein and Ambady, 2002), people do not smile only when they are joyful or amused. They also smile when they experience negative affect, such as sadness (e.g., Bonanno and Keltner, 1997; Papa and Bonanno, 2008), and they also smile in the absence of intense feelings, for example, to be polite. The purpose of the present research was to investigate whether adults of different age groups differ in how well they are able to identify such diverse emotional experiences accompanying smiles, and whether the age of the smiling persons plays a role in this respect. Below, we elucidate the theoretical background for our investigation. We first review findings from previous research on adult age differences in the ability to interpret the emotional meaning of posed facial expressions and explain methodological criticisms that have been raised regarding this line of research. We then argue that studying age differences in the ability to understand the emotional meaning of authentic smile expressions can circumvent these criticisms. We briefly introduce the state of the art of research on smile expressions, and review the available evidence from age-comparative studies on identifying different types of smiles. Following that, we explain the steps we have taken in the present studies to further advance this line of research, and derive our research questions and predictions.

### Research on age differences in identifying the emotional meaning of facial expressions: the traditional paradigm

One might expect that as adults accumulate life experience with age, they should become better at identifying the emotional meaning of other people's facial expressions. Most of the empirical evidence available to date, however, speaks to the contrary. With few exceptions, the majority of these findings stem from a paradigm that we will refer to here as the traditional paradigm: participants were presented with photographs of individuals who pose prototypic expressions of highly intense basic emotions. Their task was to select the displayed emotion from a number of response options. A meta-analysis of 17 data sets demonstrated that younger adults outperform older participants in this task (Ruffman et al., 2008). This has been shown for recognizing posed facial expressions of fear, anger, sadness, and, with smaller effect sizes, also surprise and happiness. The only exception to this overarching pattern involved the recognition of posed disgust expressions, for which no significant differences between younger and older adults emerged. Overall, however, the performance advantage of younger adults in this traditional paradigm is undisputed. These findings suggest that older adults are less adept than younger adults in recognizing emotions from facial expressions. This interpretation has recently been challenged however by various authors who pointed out important limitations in the traditional approach. Two major points of criticism have been raised, pertaining to the limited age fairness and ecological validity of the traditional paradigm (Ruffman et al., 2008; Isaacowitz and Stanley, 2011; Richter et al., 2011; Richter and Kunzmann, 2011; Riediger et al., 2011).

The first criticism, limited age fairness, argues that the selection of expression stimuli may have often put older participants at a disadvantage compared to younger participants. Most studies in this research tradition have derived their stimuli from picture sets provided by Ekman and Friesen (1976) or Matsumoto and Ekman (1988). These picture sets were selected on the basis of prototypicality judgments by younger adults, which might disadvantage older study participants if such judgments vary with age. Furthermore, these stimulus sets include facial expressions shown by younger or middle-aged, but not older, posers. This, too, might have put older participants at a disadvantage. Empirical evidence shows that people are better at interpreting emotional expressions of individuals who are similar to themselves as opposed to individuals who are dissimilar. This has been demonstrated for similarity in terms of sharing the same interests, nationality, ethnicity, cultural group, or university affiliation (Elfenbein and Ambady, 2002; Thibault et al., 2006). Several explanations for these in-group effects have been proposed, such as that one has a better knowledge base for interpreting facial expressions shown by individuals belonging to a group with which one identifies, or that one has a higher motivation to attend to, and process, the expressions of such individuals. It has been argued that age-group membership might be relevant in this regard as well, although empirical evidence to date is still rare and inconclusive (e.g., Malatesta, 1987; Ebner and Johnson, 2009; Ebner et al., 2011; Riediger et al., 2011).

The second criticism, limited ecological validity, involves the argument that spontaneous emotional expressions in “real” life differ markedly from the stimuli used in the traditional paradigm. The experimental stimuli typically showcase photographs of prototypical displays, as proposed by the Affect Program Theory of facial expressions (APT, e.g., Ekman, 1972, 1993). Evidence is amounting, however, that facial expressions that are spontaneously shown while experiencing emotions are often more subtle, and typically comprise the activation of fewer and sometimes different muscular components (action units) than proposed by APT, even when the self-rated intensity of the emotional experience is high (for a review, see Reisenzein et al., 2013).

Another characteristic of the traditional paradigm that delimits its ecological validity is that still pictures of facial expressions are used as experimental stimuli. The spontaneous facial expressions that people encounter in their daily lives, however, are dynamic and rapidly changing. Evidence that partly different brain structures subserve the processing of static and dynamic stimuli underscores that the temporal dimension is an important characteristic of facial expressions (e.g., Adolphs et al., 2003; LaBar et al., 2003; Schultz and Pilz, 2009).

Like limited age fairness, the limited ecological validity may also have disadvantaged older adults more than younger adults because solving tasks that have not been practiced before appears to be more challenging for older than for younger adults. Age differences in various types of cognitive performance, for example, have been found to be more pronounced for unfamiliar or artificial problems and considerably smaller for ecologically valid problems that older adults encounter in their daily life contexts (e.g., Phillips et al., 2006; Kliegel et al., 2007). It seems reasonable to expect that this effect would generalize to differences between artificial and ecologically valid emotion-recognition tasks as well.

In essence, research on whether adults from different age groups differ in their ability to identify the emotional experiences accompanying facial expressions has a long tradition. The paradigm that has predominantly been used to date to investigate this question, however, has been criticized for various reasons. These criticisms have inspired researchers to search for alternative paradigms. One novel approach, which we consider promising and pursue further in the research reported here, is to investigate whether adults from various age groups differ in how well they are able to identify the emotional experiences accompanying smile expressions that were displayed in different emotional situations (McLellan, 2008; Murphy et al., 2010; Slessor et al., 2010a). Another approach to enhance ecological validity has been to study age differences in emotion recognition in naturalistic (e.g., Rauers et al., 2013) or semi-naturalistic situations (e.g., Richter and Kunzmann, 2011; Sze et al., 2012). In such situations, multiple information sources can be available (e.g., previously acquired knowledge about the interaction partner, the content of verbal communication, and other channels of non-verbal communication in addition to facial expressions, like pitch of voice, body posture and -movements). Therefore, this alternative research approach is well suited to examine age differences in the overarching ability to understand other people's emotional experiences—an ability that derives from integrating information from these multiple sources. In these studies, however, it is difficult to separate the specific components that contribute to this integrative ability. One of these specific skills is in the fore of the present paper: the ability to infer emotional states specifically from facial expressions.

### Identifying emotional experiences that accompany smile expressions

Smiles are subtle facial displays that frequently occur in natural interactions. The smile expression involves lifting the corner of the mouth through contraction of the zygomaticus major muscle. It can, but need not, be accompanied by activation of other facial muscles as well (e.g., Ambadar et al., 2009). Smiling differs from laughing in that neither the characteristic laughing acoustic nor the typical laughing movements (e.g., rhythmically repeated head and shoulder movements) accompanies it.

Smile expressions are shown in different emotional situations. People often smile in positive emotional contexts, for example, when they feel happy or amused. In fact, smiling is regarded as a prototypical sign of pleasure in many cultures (e.g., Ekman, 1972; Elfenbein and Ambady, 2002). People also however occasionally smile in the absence of pleasant feelings, and even in the context of negative emotional experiences, such as feeling embarrassed (e.g., Keltner, 1995) or sad (Bonanno and Keltner, 1997; Papa and Bonanno, 2008). It has been argued that smiling in the absence of positive affect serves self-regulatory as well as social functions (Gross, 1999). It has been found, for example, that smiling can help to alleviate negative and enhance positive affect (Soussignan, 2002; Ansfield, 2007). People may also smile in the absence of positive affect to conform to social norms and expectations, for example, to conceal how they are feeling, or to appease their interaction partner (e.g., Keltner, 1995; Hecht and LaFrance, 1998; Keltner and Haidt, 1999).

The smile displays that accompany different emotional experiences are assumed to vary subtly in their expressive characteristics. What exactly these expressive differences are, is subject of an ongoing debate. Two types of smiles have been most extensively investigated in this regard, namely, those that are spontaneously shown when experiencing positive emotional states, and those that are deliberately posed in the absence of positive experiences (for overviews, see Ambadar et al., 2009; Johnston et al., 2010). An assumption that is still widely spread, but critically questioned today, traces back to the 19th century French physiologist Duchenne de la Boulogne. It pertains to the lateral part of the muscle surrounding the eye (i.e., the pars lateralize of the orbicularis oculi muscle, also referred to as the Duchenne marker), which has been proposed to contract during spontaneous smiles (thus narrowing the eyes and creating characteristic crow's feet), but not during deliberate smiles (e.g., Ekman et al., 1990; Soussignan, 2002). Empirical evidence, however, contradicts the assumption that the Duchenne marker indicates spontaneous, positive-affect smiles. Neither do positive-affect smiles reliably involve the presence, nor do non-positive smiles reliably involve the absence of the Duchenne marker (see overviews in Messinger et al., 1999; Krumhuber and Manstead, 2009). That is, contraction of the Duchenne marker also occurs at a considerable number of occasions when people deliberately pose smiles (Schmidt and Cohn, 2001; Schmidt et al., 2006, 2009; Krumhuber and Manstead, 2009), or when they smile while experiencing negative affect, such as embarrassment (e.g., Keltner, 1995; Papa and Bonanno, 2008). Also the proposal that spontaneous positive-affect smiles may be more symmetrical than posed smiles (e.g., Ekman et al., 1981) has not been supported by studies using precise measures of expression symmetry (e.g., Schmidt et al., 2006). Instead, evidence is accumulating that types of smiles may differ in their temporal characteristics (see overview in Schmidt et al., 2009). Compared to posed smiles, for example, spontaneous positive smiles have been found to involve longer onset and offset phases (Schmidt et al., 2006), and a smoother progression of muscle movements (Hess and Kleck, 1990). Overall, there is considerable agreement among smile researchers that there are expressive differences between smiles accompanying different emotional experiences. The exact nature of these expressive differences, however, still is an ongoing field of research.

Let us return to the question of whether adults from different age groups differ in their ability to identify emotional experiences accompanying facial expressions. One novel approach in this line of research, taken so far by three studies, has been to use the smile expressions that had been shown in different emotional situations as the experimental stimuli (as opposed to posed expressions of highly intense basic emotions in the traditional paradigm). Adults from different age groups were asked to choose among several response options the emotional context in which they believed that the specific smile expression had been shown (McLellan, 2008; Murphy et al., 2010; Slessor et al., 2010a). As described in more detail below, these studies suggest that the conclusion from studies with the traditional paradigm (namely, that the ability to identify emotions from facial expressions uniformly declines with age) needs to be modified. This novel approach is interesting because it circumvents shortcomings of the traditional paradigm, yet still maximizes experimental control: smile expressions are highly relevant in people's everyday life contexts and hence the requirement of ecological validity discussed above is fulfilled. In addition, smiles occur in different emotional contexts and therefore lend themselves as stimuli for the study of individual differences in the ability to identify emotional experiences accompanying facial expressions. Smile expressions thus do indeed appear to be well suited as experimental stimuli in studying adult age differences in the ability to identify emotional facial expressions. The smiles studies available to date, however, have had some limitations so that further evidence is necessary. The above review indicates that several requirements need to be met to fully exploit the potential advantages of using smile expressions as experimental stimuli. First, given the potential diagnostic value of the temporal characteristics in identifying the emotional experiences accompanying smile expressions, the smile stimuli should be dynamic rather than static. Second, because the research on mimic characteristics that reliably indicate the emotional experience during a smile episode is still evolving, the smiling persons' emotional experiences should not be determined on the basis of expression characteristics. In particular, the Duchenne criterion should be avoided, given accumulating evidence that it does not reliably differentiate between different types of smiles. An alternative approach to maximizing the content validity of smile types could be, for example, to establish that the smiling persons' self-reported emotional experiences correspond to the emotional nature of the situations in which they displayed the smile. Third, in light of previous evidence on in-group advantages in understanding emotional facial expressions, the age of the smiling person should be varied to enhance the age fairness of the task and to determine the role that the age of the smiling person plays for participants' recognition accuracy. Fourth, the range of emotional experiences that accompany the selected smile expressions should be wide and ideally include positive, negative, as well as emotionally neutral states. This latter stipulation is in line with the claim that to understand adult age differences in various aspects of emotional functioning, it is important to consider the valence dimension of emotional experiences. This claim derives from the assumption that as older adults become increasingly aware of their narrowing perspective of remaining life time, they should become progressively more motivated to optimize their emotional well-being in the here and now. This, in turn, should be reflected in an increasing preference to attend to and process positive rather than negative information from their surroundings (e.g., Carstensen et al., 2003). Indeed, there is ample evidence to support this claim, primarily in the domains of attention and memory (for overviews, see Carstensen and Mikels, 2005; Reed and Carstensen, 2012), but also in the ways how adults from different age groups interpret still pictures of emotional poses as used in the traditional paradigm (Riediger et al., 2011). It is possible that these preferences might also influence how individuals from different age groups interpret the emotional meaning of different type of smiles.

To the best of our knowledge, no previous study has been published thus far that fulfilled all of these requirements. We are aware of three previous publications on adult age differences in reading different types of smiles (McLellan, 2008; Murphy et al., 2010; Slessor et al., 2010a). All of these studies were interested in the ability to differentiate spontaneous, positive-affect smiles from posed, emotionally neutral smiles. None of these studies included smile expressions accompanying negative emotional experiences. The results of these studies indicated either no significant differences between younger and older adults in the ability to differentiate smile expressions (McLellan, 2008; Murphy et al., 2010, Study 1; Slessor et al., 2010a), or better performance in older as compared to younger adults (Murphy et al., 2010, Study 2). McLellan (2008) and Slessor et al. (2010a) used still pictures of smile expressions and thus did not provide the potentially diagnostic information of the smile dynamics. Furthermore, they included smile expressions only from young, but not older target persons, and used the Duchenne marker as a selection criterion for their smile stimuli, in addition to the targets' self-reported emotional experiences. Murphy et al. (2010) did use dynamic smile stimuli and also varied the age of the smiling person in one of the reported studies. They relied, however, exclusively on the Duchenne criterion to categorize their smile stimuli as positive-affect vs. posed smiles, without further verifying this, for example, with the smiling persons' self-reported experience or information on the emotional nature of the situation in which the smile was shown.

The purpose of the present research was to further advance this line of research by creating a content-valid set of dynamic smile stimuli that fulfills all of the requirements summarized above. This set of dynamic stimuli comprised smile expressions that were spontaneously displayed while experiencing elevated levels of either positive or negative affect, or that were displayed upon our request while being in an emotionally neutral state. We employed these stimuli in two studies. In Study 1, positive, negative, and neutral smile expressions displayed by younger targets were presented to younger and older adults. The participant's task was to identify the emotional experience accompanying the presented smile expressions. Because of their accumulated exposure to, and experience with, subtle emotional expressions of other persons, we expected older participants to be more accurate in their performance of this ecologically and content-valid smile emotion-recognition task than younger adults would be. We also explored whether this age effect would differ between positive-affect, negative-affect, and posed smiles. This exploration was motivated by prior evidence of age-related increases during adulthood in preferential attention toward positive and away from negative information (Carstensen and Mikels, 2005; Reed and Carstensen, 2012). We were interested in exploring whether these positivity effects would generalize to interpretations of smile expressions as well.

In Study 2, we presented positive and neutral smile expressions of younger and older targets to younger and older adults. Our aim was to investigate whether the age of the smiling person matters for the perceiver's accuracy in identifying emotional experiences accompanying smiles. Previous research suggests that people are better able to identify emotional experiences from facial expressions shown by individuals that belong to the same social group, broadly defined (Elfenbein and Ambady, 2002). We hypothesized that age-group membership may have similar effects on identifying the emotional meaning of dynamic smile expressions shown by target persons from different age groups. We are aware of only one prior study that investigated this possibility (Murphy et al., 2010). There was no indication of own-age effects in smile recognition in this study. This, however, could have been because the smile expressions in this study had been classified exclusively on the basis of the Duchenne criterion (i.e., without reference to the situations in which the smiles were shown or to the smiling persons' self-reported emotional experience). Because of previous evidence that the Duchenne marker is not a reliable indicator of emotional experiences accompanying smile expressions, we assumed that a different pattern might emerge in our study, which used smile stimuli that had been selected when the smiling person's self-reported emotional experience and the emotional nature of the situation in which the smile had been expressed corresponded with one another.

## Pre-study: stimulus development

To fulfill the four requirements introduced above, we developed a new set of dynamic and content-valid smile expressions that were displayed in different emotional situations by target persons varying in age and gender whose self-reported emotional experiences matched the intended emotional context. The collection included negative-affect, positive-affect, and emotionally neutral smiles. The videos recorded the head and shoulders of the target persons. They started with the onset of the smile expression, ended with its offset, and did not include sound. Their average duration was 6.99 s (*SD* = 2.27). Smile duration differed neither between the three smile types, *F*_(2, 77)_ = 0.031, *p* = 0.970, partial η^2^ = 0.001, nor between the two age groups of target persons, *F*_(1, 77)_ = 0. 385, *p* = 0.537, partial η^2^ = 0.005, and there was also no significant Smile Type × Age Group interaction, *F*_(1, 77)_ = 0.131, *p* = 0.718, partial η^2^ = 0.002.

Below, we describe the development and selection of these smile stimuli and demonstrate that the target persons' self-reported emotional experiences during the smile episodes matched the intended emotional situation and differed significantly between the three types of smiles. We also report the prevalence rates of the Duchenne marker, which confirm prior evidence that this marker is not suited to distinguish between different types of smiles.

### Selection of negative-affect smiles displayed by younger target persons

Negative-affect smiles were extracted from video-recordings of a previously published anger-induction experiment with *N* = 157 non-psychology majors of the University of Greifswald, Germany, who had signed up to participate in a study allegedly investigating associations between personality and concentration (Weber and Wiedig-Allison, 2007). Of these participants, 34 persons were excluded because they reported suspicions during debriefing about the true study purposes, leaving recordings of 123 persons (60 female, *M* = 22.9 years of age, *SD* = 3.0) as material for the extraction of negative-affect smiles. Prior to the anger-induction phase, these target persons first completed, among other things, baseline measures of state anger and momentary negative affect. They were then instructed to work on a computerized task, and to only use certain keys for their responses. The task was programmed to break down after several minutes and trained experimenters accused the target persons of having caused the breakdown by pressing a wrong key. The experimenters also commented in a brusque and condescending way on the situation, implying that the participants had failed on a very simple task. After pretending that restarting the task had failed, the experimenter announced that the data had been lost due to the target person's fault and that he or she might therefore not receive the promised reimbursement. Following that, the target persons again completed, among other things, measures of momentary state anger and negative affect. Then they were debriefed about the true nature of the experiment and asked whether they still consented to be part of the study and whether they would permit their videotapes to be used in further studies and analyses (which all target persons did).

State anger was measured before and after the anger induction phase using four items from the German version of the State Anger Scale (Schwenkmezger et al., 1992). Items were responded to on a four-point rating scale. A sum score was determined as an indicator of the target person's momentary state anger and rescaled such that absence of anger was indicated by a value of zero. Negative affect was assessed with 10 items from the German version of the Positive and Negative Affect Schedule (Krohne et al., 1996), which were responded to on a five-point rating scale. A sum score of these items served as an indicator of momentary negative affect and was rescaled such that absence of negative affect was indicated by a value of zero.

In the video recordings, smile episodes during the anger-induction phase were identified as contractions of the zygomaticus major muscle (lip corner puller AU12 in the Facial Action Coding System FACS, Ekman and Friesen, 1978). From the resulting pool of 72 smile episodes, 16 negative-affect smiles (50% from female target persons) were selected that fulfilled the following criteria: (1) The smile was shown following insulting remarks by the experimenter; (2) The smile was displayed by target persons with an increase in momentary negative affect or an increase in state anger from baseline to post-induction, respectively; and (3) The smile expression was complete (i.e., included onset, apex, and offset phases) and unambiguous (i.e., included no more than one contraction of the zygomaticus major muscle). The average momentary state anger and the average momentary negative affect reported by the respective target persons after the anger induction phase are summarized in the first row of Table 1 for the negative-affect smiles that were selected.

**Table 1:** **Self-reported emotional experiences accompanying the negative-affect, positive-affect, and neutral smile episodes selected as stimulus material for Studies 1 and 2**.

**Smile type**	**Age group of target**	**State anger^a^**		**Negative affect^a^**		**Amusement^b^**	
		***M* (POMP^c^)**	***SD***	***M* (POMP^c^)**	***SD***	***M* (POMP^c^)**	***SD***
Negative-affect smile	Younger	3.75 (31.25%)	2.82	12.69 (31.73%)	6.99	–	–
	Older	–	–	–	–	–	–
Positive-affect smile	Younger	0.00 (0.00%)	0.00	0.69 (1.73%)	1.25	80.42 (80.42%)	12.46
	Older	0.00 (0.00%)	0.00	0.94 (2.35%)	1.06	79.38 (79.38%)	12.06
Neutral smile	Younger	0.00 (0.00%)	0.00	0.69 (1.73%)	1.14	10.83 (10.83%)	8.48
	Older	0.00 (0.00%)	0.00	0.38 (0.95%)	1.09	15.00 (15.00%)	10.11

*Sixteen smile episodes per smile type and age group of targets*.

a *State anger and negative affect were assessed after the anger-induction phase, amusement-induction phase, and posing-instruction phase for negative-affect, positive-affect, and neutral smiles, respectively*.

b *Amusement was assessed immediately after each smile episode*.

c *POMP, percent of maximum possible score*.

### Selection of positive-affect and neutral smiles displayed by younger and older target persons

Episodes of positive-affect and neutral smiles were recorded at the Max Planck Institute for Human Development in Berlin during an amusement-induction phase that was followed by a posing-instruction phase. The setting meticulously mirrored that of the negative-affect smile recordings with regard to furniture, background, and lighting. Contrast, depth of focus, brightness, and light temperature of the camera recordings were also matched to the negative-affect smile recordings, as were the clothing and movements of the target persons.

The sample of target persons included 42 younger adults (*M* = 23.64 years, *SD* = 2.36, 21 female), and 48 older adults (*M* = 74.25, *SD* = 3.37, 22 female). All target persons were residents of the Berlin area, Germany, and were recruited via the participant database of the Max Planck Institute for Human Development, Berlin. After giving their informed consent, they practiced various head and body movements that re-enacted those shown during the negative-affect smile interactions, where target persons had rarely sat still, but often had looked toward or away from the insulting experimenter. To ensure comparability between the three smile types with regard to the accompanying movements, we instructed the target persons to re-enact these movements (e.g., “Move your head to look at the red dot on the wall.”). These instructed movements were practiced further while four emotionally neutral video clips were being watched. Following that, the target persons completed, among other things, baseline measures of momentary state anger and negative affect, using the same instruments as in the anger-induction experiment. Then, the target persons were video-recorded while watching funny video-clips (*n* = 10) and cartoons (*n* = 5) and performing the practiced movements upon request. Of the amusement stimuli, eleven were shown to all target persons, whereas four (3 cartoons and 1 clip) were selected to accommodate assumed differences in what younger and older persons might consider funny and thus differed between the two age groups. Following each amusement stimulus, target persons rated how amused, exhilarated, and cheerful they momentarily felt on a scale ranging from 0 (not at all) to 100 (very much). A mean score of these items served as an indicator of momentary amusement. Target persons also indicated after each amusement stimulus whether they had experienced any other feeling besides amusement. If yes, they reported the respectively most dominant feeling in an open response format (which was later content-coded by two independent coders) and rated its intensity on a scale from 0 to 100. This open-response item was included to ensure that the affective experience accompanying the smile expression was unequivocal in valence (and not, for example, also accompanied by feelings of awkwardness due to the experimental situation or the instructed movements). Following the amusement induction phase, participants again reported their momentary state anger and negative affect.

After that, participants were instructed to put themselves into an emotionally neutral state as much as possible. The experimenter described six to ten situations in which people smile without experiencing intense positive or negative feelings (e.g., “Imagine a friend telling a joke that you find neither funny nor juicy. To be polite, you smile.”) The target persons' task was to display the smile expression they would show in such a situation and to also perform the movements practiced before upon request. The recording of the neutral smile expressions was scheduled last because, to do the recordings, we had to reveal our interest in smile expressions and we wished to avoid that the target persons' awareness of our interest in smile expressions might affect the genuineness of the positive-affect smiles they displayed while watching the amusing material.

After each posed smile, the target persons again rated their momentary amusement and potential other feelings, using the same items as in the amusement-induction phase. The experimenter ended the posing phase after six to ten posing attempts, when at least one suitable smile expression had been recorded. Following that, the target persons again rated their momentary state anger and negative affect.

Smile episodes during the amusement induction and posing instructions were identified from the video recordings as contractions of the zygomaticus major muscle (AU12). From the resulting pool of smile episodes, 16 positive-affect smiles from younger target persons (50% female) and 16 positive-affect smiles from older target persons (50% female) were selected that fulfilled the following criteria: (1) The smile was spontaneously shown in response to an amusing stimulus; (2) The smile was displayed by target persons (a) who reported intense amusement (at least 60 on a scale from 0 to 100) and no other feelings, and (b) who reported low negative affect (no more than 4 on a scale from 0 to 40) and no state anger after the amusement-induction phase; and (3) The smile expression was complete (i.e., included onset, apex, and offset phases) and unambiguous (i.e., included no more than one contraction of the zygomaticus major muscle).

We also selected 16 neutral smiles from younger target persons (50% female) and 16 neutral smiles from older target persons (50% female) according to the following criteria: (1) The smile was displayed in response to the experimenter's instruction to pose a smile; (2) The smile was displayed by target persons who reported little amusement (no more than 30 on a scale from 0 to 100) and no additional feelings after the smile episode, and who reported low negative affect (no more than 4 on a scale from 0 to 40) and no state anger after the posing-instruction phase; and (3) The smile expression was unambiguous, included onset, apex, and offset phases, but did not involve multiple contractions of the zygomaticus major muscle.

The second and third rows in Table 1 summarize, for the positive-affect and neutral smiles chosen, the average momentary state anger and negative affect reported by the target persons after the amusement-induction and posing-instruction phases, respectively, and the average amusement reported immediately after the selected smile episodes.

### Manipulation check: differences between smile types in relation to target persons' accompanying emotional experiences

To statistically substantiate that the selected episodes for the three types of smiles significantly differed with regard to the targets' self-reported emotional experiences, we first compared the positive-affect and neutral smile episodes expressed by younger and older target persons. A multivariate analysis of variance on negative affect (reported after the amusement induction and the posing instruction, respectively) and amusement (reported immediately after each selected smile episode) confirmed a significant multivariate effect of smile type, *F*_(2, 59)_ = 1298.12, *p* = 0.000, partial η^2^ = 0.91, according to Wilks Lambda, whereas neither the main effect of age group of target, *F*_(2, 59)_ = 0.17, *p* = 0.847, partial η^2^ = 0.01, nor the Age Group × Smile Type interaction, *F*_(2, 59)_ = 0.92, *p* = 0.405, partial η^2^ = 0.03, reached statistical significance. (State anger was not included in this analysis because none of the targets reported any anger experiences for the positive and neutral smile episodes.) A multivariate main effect of smile type also emerged when we compared negative, positive, and neutral smile expressions by younger targets with regard to state anger and negative affect, *F*_(4, 88)_ = 16.24, *p* = 0.000, partial η^2^ = 0.43, according to Wilks Lambda. (Older targets were not included in this analysis because no negative-affect smiles were available for them; amusement ratings were not included because they were not available for negative-affect smiles).

Pairwise comparisons confirmed that targets had experienced significantly higher state anger and negative affect when displaying negative-affect smiles than when displaying positive-affect smiles [state anger: *T*_(15)_ = 3.75, *p* = 0.000; negative affect: *T*_(15.41)_ = 6.75, *p* = 0.000] and neutral smiles [state anger: *T*_(15)_ = 5.33, *p* = 0.000; negative affect: *T*_(15.38)_ = 6.91, *p* = 0.000]. Positive-affect and neutral smiles did not differ with regard to state anger and negative affect (all *p* > 0.05). Compared to neutral smiles, however, positive-affect smiles were accompanied by significantly more intense amusement, *T*_(62)_ = 24.74, *p* = 0.000.

Overall, these analyses indicate that the aims of the stimulus development were met. The three smile types differed significantly in the target persons' self-reported emotional experiences, which in turn matched the intended emotional nature of the situation in which the smile expression was shown. There was no indication that this effect differed between younger and older target persons.

### Duchenne coding of smile selection

The third author rated the presence/absence of the Duchenne marker (AU6, Ekman and Friesen, 1978) for the selected smile episodes. Coding was instructed and supervised by the second author who is an experienced FACS coder. Coding agreement with a second independent coder was satisfactory (across all smile episodes: κ = 0.91; across smile episodes from younger targets: κ = 0.95; across smile episodes from older targets: κ = 0.76; both coders were young adults). Of the 80 smile episodes, 10 (2 negative-affect smiles, 4 positive-affect smiles, and 4 neutral smiles) could not be reliably coded because hair or glasses partially obscured the eye region. Activation of the Duchenne marker was present in 57.1% of the remaining negative-affect smiles (younger targets only), in 57.1% of the remaining positive-affect smiles shown by younger targets; in 71.4% of the remaining positive-affect smiles shown by older targets; in 35.7% of the remaining neutral smiles shown by younger targets; and in 92.9% of the remaining neutral smiles shown by older targets. These findings are consistent with previous evidence that the Duchenne marker does not reliably distinguish between different emotional experiences accompanying smile expressions (see overviews in Messinger et al., 1999; Krumhuber and Manstead, 2009).

## Study 1

### Methods

#### Sample

The sample consisted of *N* = 100 participants living in or around Berlin, Germany. Participants were recruited via a participant database of the Max Planck Institute for Human Development. Requirements for study participation were (a) that the participants were either between 20 and 30 years of age (*n* = 48, *M* = 25.75, *SD* = 2.61), or between 70 and 80 years of age (*n* = 52, *M* = 74.53, *SD* = 3.05); (b) that their mother tongue was German, (c) that they had sufficient (corrected) vision to see videos presented on a computer screen clearly; and (d) that they had not taken part in the Pre-Study for stimulus development. Both age groups were approximately stratified by gender (50 and 46.2% female in the younger and older age groups, respectively) and education (54.2% and 40.4% with German university-entrance qualification in the younger and older age groups, respectively). Informed consent was obtained from all participants, and the ethics committee of the Max Planck Institute for Human Development had approved of the study.

#### Measures

***Smiles task***. Participants watched 48 videos of positive-affect smiles (*n* = 16), negative-affect smiles (*n* = 16), and neutral smiles (*n* = 16) expressed by younger targets. The presentation sequence of the videos was randomized. Prior to each video, participants saw a still picture of the start frame for 3 s, showing the target with a neutral expression that preceded the onset of the smile. It was presented so that participants could acquaint themselves with the physiognomy of the target before the smile task. After each video, participants completed the sentence stem “The person smiled in a situation …” by selecting one of three response options: (a) “in which he or she experienced a pleasant feeling (e.g., amusement),” (b) “in which he or she experienced an unpleasant feeling (e.g., anger),” or (c) “in which he or she posed a smile without feeling anything.” For the sake of simplicity, we will refer to these response categories as positive-affect, negative-affect, and neutral smiles below. The smiles task was programmed in DMDX (Forster and Forster, 2003).

Accuracy of categorization was determined as unbiased hit rate, following the procedure proposed by Wagner (1993). This measure accounts for the potentially distorting effects of response tendencies (which can be illustrated by the example of a blindfolded person who would obtain an uncorrected hit rate of 100% in one category without even looking at the stimuli if he always chose the same response). The unbiased hit rate is the joint probability that a smile type was correctly identified and that the response option was correctly used, and is thus insensitive to such a bias in responding. It is determined as the product of the conditional probability that a given smile type was correctly identified (i.e., the number of correctly identified stimuli from that smile type divided by the total number of stimuli from that smile type) and the conditional probability that a response category is correctly used, given that it is used (i.e., the number of correct uses of a response category divided by the total number of uses of that response category).

***Control variables***. As control variables in the present research, we included measures of participants' education, as well as of crystallized cognitive, fluid cognitive, and visual abilities, all of which have been discussed as possible mechanisms that might underlie age-related differences in emotion recognition (e.g., Phillips et al., 2002; Keightley et al., 2006; Murphy et al., 2010). More specifically, participants indicated their *years of education* for the number of school years completed and the number of years in professional training. A sum score indicating the total number of years of education was used as a covariate. *Perceptual-motor speed* was measured using the Digit-Symbol Substitution Test of the Wechsler Intelligence Scales (Wechsler, 1981). Participants were given mappings of symbols and digits, and their task was to draw the corresponding symbols for a series of digits as fast as possible. The number of correct responses completed within 90 s served as an indicator of perceptual-motor speed. Participants' *vocabulary* was assessed with a test in which participants had to find real words among various pseudo-words (MWT-A, Lehrl et al., 1991). Participants' vision was assessed with two subtests of the computerized Freiburg Visual Acuity and Contrast Test (FrACT, Bach, 2007). In the Acuity Landolt C subtest of the FrACT assessing *visual acuity*, participants were presented with a series of 24 Landolt rings (i.e., rings that have a gap, thus looking similar to the letter C) varying in size. The position of the gap varied across stimuli. The participant's task was to choose, via button press, the gap's correct position out of eight possibilities. The size of the Landolt rings was varied depending on the participant's performance, and visual acuity was determined as decimal acuity (Snellen's fraction; higher values indicate better visual acuity). *Contrast sensitivity* was measured as the Michelson contrast using the subscale Contrast of the FrACT in which participants were presented with a series of 24 Landolt rings that varied in their luminance (smaller values indicate better contrast sensitivity).

In Table 2 these control variables are compared for the younger and older subsamples. Compared to older participants, younger participants reported more years of school education, obtained higher scores in the perceptual-speed task, lower scores in the vocabulary task, and had better visual acuity and contrast sensitivity. Younger and older participants did not differ significantly with regard to years of professional training. Overall, this pattern of age-group differences is consistent with what is to be expected based on the developmental literature.

**Table 2:** **Descriptives of control variables in Study 1**.

**Construct**	**Younger participants (perceiver)**		**Older participants (perceiver)**				
	***M***	***SD***	***M***	***SD***	***F***	***df***	***p***
Years of school education	12.33	1.55	10.98	2.21	11.860	1, 96	0.001
Years of professional training	3.60	2.00	5.04	5.84	2.477	1, 94	0.119
Perceptual-motor speed (Digit-Symbol)	61.90	11.36	41.67	7.69	110.075	1, 98	0.000
Vocabulary knowledge (MWT-A)	28.94	2.95	32.52	1.75	55.462	1, 98	0.000
Visual acuity (decimal acuity)	1.47	0.28	0.83	0.39	87.808	1, 98	0.000
Contrast sensitivity (Michelson contrast)^a^	0.66	0.34	1.94	1.20	50.416	1, 98	0.000

*Multivariate age group effect according to Wilks Lambda: F_(6, 87)_ = 41.68, p = 0.000, partial η^2^ = 0.74*.

a *Smaller values indicate better contrast sensitivity*.

### Results

In the following, we first report analyses of potential age-related differences in participants' response tendencies (irrespective of whether the responses were correct). Then we report age differences in the unbiased hit rates for identifying emotional experiences accompanying smiles and analyze whether the observed unbiased hit rates differed significantly from chance.

#### Response tendencies

**Figure 1:**
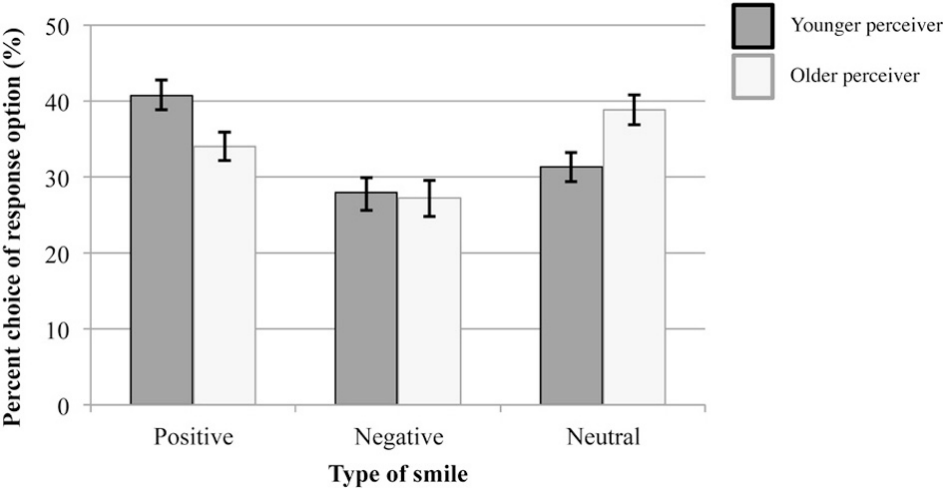
**Use of response options in Study 1 (irrespective of whether response choice was correct)**. Error bars represent ±1 standard errors from the mean.

Figure 1 shows the percentages with which younger and older participants chose each of the three response options for evaluating the emotional experience accompanying smiles, irrespective of whether or not the chosen responses were correct. Results of a multivariate analysis of variance on the percentage of chosen responses with age group (younger and older participants) as between-person factor, and response option (positive-affect smile, negative-affect smile, neutral smile) as within-person factor are summarized in Table 3. The significant interaction of Age Group × Response Option indicates younger and older participants varied in their response preferences. This underscores the importance of using the unbiased hit rate (Wagner, 1993) when analyzing how well participants from different age groups were able to identify emotional experiences accompanying smiles. Follow-up analyses of this interaction showed that in comparison to younger adults, older adults evaluated smile expressions less frequently as being accompanied by positive affect, *F*_(1)_ = 5.84, *p* = 0.018, partial η^2^ = 0.06, and more frequently as being emotionally neutral, *F*_(1)_ = 7.64, *p* = 0.007, partial η^2^ = 0.07. Younger and older adults did not differ in the percentage with which they judged smile expressions as being accompanied by negative affect, *F*_(7)_ = 0.07, *p* = 0.799, partial η^2^ = 0.001. Comparisons within age-group showed that younger adults chose the response option positive-affect smile significantly more frequently than each of the other two response options [negative-affect smile: *F*_(1)_ = 13.00, *p* = 0.001, partial η^2^ = 0.22; neutral smile: *F*_(1)_ = 7.49, *p* = 0.009, partial η^2^ = 0.14]. Their percentages of choosing the response options negative-affect smile and neutral smile did not differ significantly (*p* > 0.05). In contrast, older adults chose the response option neutral smile significantly more often than the response option negative-affect smile, *F*_(1)_ = 8.55, *p* = 0.005, partial η^2^ = 0.14. All other pairwise comparisons of older adults' percentages of chosen response options were non-significant (all *p* > 0.05).

**Table 3:** **Predicting use of response options (irrespective of whether response was correct) in Study 1**.

**Effect**	***F***	***df***	***p***	**Partial eta squared**
Response option^a^	7.331	2, 97	0.001	0.131
Response option × Age group of perceiver^a^	4.901	2, 97	0.009	0.092
Age group of perceiver	0.055	1	0.816	0.001

a *Multivariate F-test based on Wilks Lambda*.

#### Age differences in unbiased hit rate of identifying emotional expressions accompanying smiles

**Figure 2:**
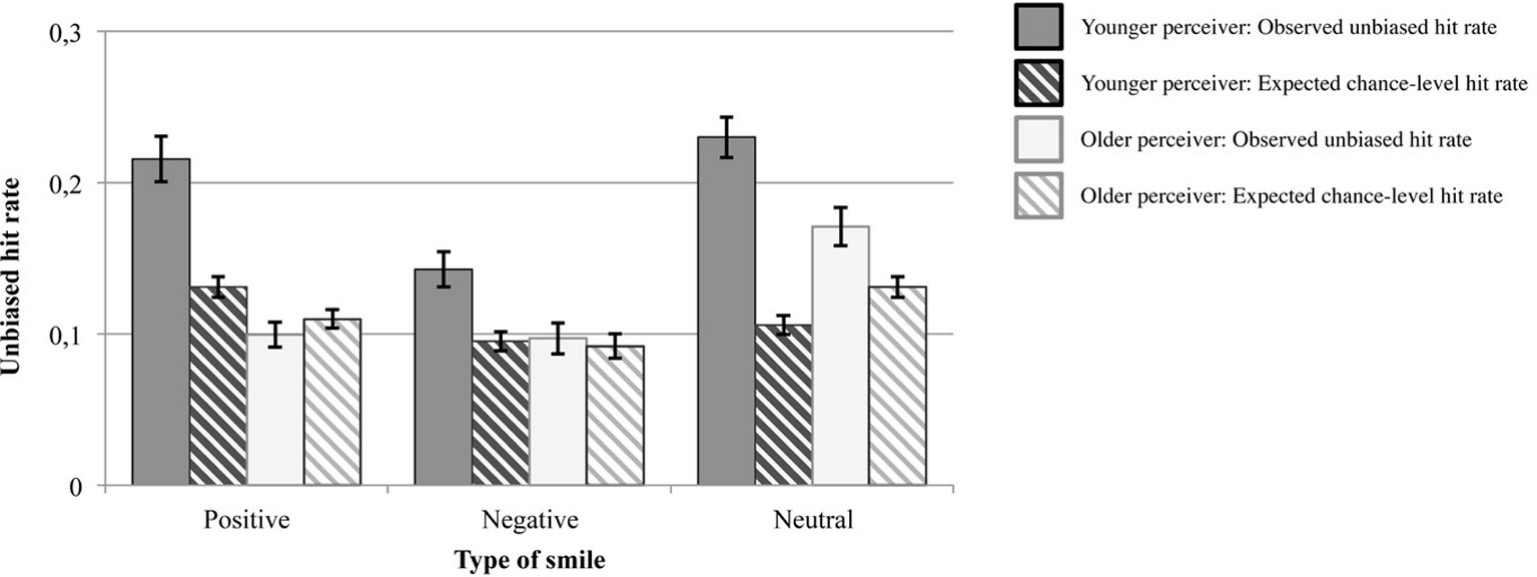
**Observed unbiased hit rates and expected chance-level hit rates in Study 1**. Error bars represent ±1 standard errors from the mean.

Solid bars in Figure 2 represent the average unbiased hit rates of correctly identifying positive, negative, and neutral smiles for younger and older participants. Striped bars indicate the average expected chance levels of performance. In the following, we first analyze age-related differences in unbiased hit rates, and then investigate whether the observed unbiased hit rates were significantly different from chance-level performance.

Results of a multivariate analysis of variance with age group of participants (younger and older) as between-person factor, and smile type (positive-affect smile, negative-affect smile, neutral smile) as within-person factor on unbiased hit rates are summarized in Table 4. Particularly important for our hypotheses is the significant main effect for age group of participant, which was qualified by the significant interaction of Age Group × Smile Type. Follow-up analyses on this interaction indicated that younger participants, contrary to our prediction, outperformed older participants in correctly identifying the accompanying emotional experiences for all three types of smile [positive-affect smiles: *F*_(1)_ = 47.96, *p* = 0.000, partial η^2^ = 0.33; negative-affect smiles: *F*_(1)_ = 8.59, *p* = 0.004, partial η^2^ = 0.08; neutral smiles: *F*_(1)_ = 10.27, *p* = 0.002, partial η^2^ = 0.10], and this age difference was most pronounced for positive-affect smiles (see Figure 2)^1^.

**Table 4:** **Predicting unbiased hit rates in Study 1**.

**Effect**	***F***	***df***	***p***	**Partial eta squared**
Type of smile^a^	22.674	2, 97	0.000	0.319
Type of smile × Age group of perceiver^a^	5.545	2, 97	0.005	0.103
Age group of perceiver	49.519	1	0.000	0.336

a *Multivariate F-test based on Wilks Lambda*.

The central finding of this analyses—the main effect of age group of participants remained—significant when we controlled for participants' years of education, processing speed, vocabulary knowledge, visual acuity, and visual contrast sensitivity, *F*_(1)_ = 14.67, *p* = 0.000, partial η^2^ = 0.14. The main effect of type of smile as well as the interaction of Age Group × Smile Type no longer reached significance in this control analysis, *F*_(2, 92)_ = 0.70, *p* = 0.502, partial η^2^ = 0.02, and *F*_(2, 92)_ = 2.27, *p* = 0.109, partial η^2^ = 0.05, according to Wilks Lambda, respectively.

In a next step, we followed the procedure proposed by Wagner (1993, p. 18 f.) to investigate whether the observed unbiased hit rates differed significantly from chance-level performance. We first determined, separately for each participant, the unbiased hit rates that were to be expected by chance for each of the three smile types, given the participants' use of response options (i.e., we determined the probability with which a given participant would choose a correct response option by chance when a particular smile type was presented). This was achieved by multiplying the relative frequency of a given smile type (among all smile stimuli) with the relative frequency with which a given participant had chosen the corresponding response option (among all responses). The average resulting chance-level probabilities for correct responses are depicted as striped bars in Figure 2. To investigate statistically whether the observed unbiased hit rates were significantly different from these chance-level probabilities, we specified a multivariate analysis of variance with age group (younger and older participants) as between-person factor, and smile type (positive-affect smile, negative-affect smile, neutral smile) as well as type of hit rate (observed unbiased hit rate and chance-level hit rate) as within-person factors. This analysis yielded a significant three-way interaction of Age Group × Smile Type × Type of Hit Rate, *F*_(2, 97)_ = 4.54, *p* = 0.013, partial η^2^ = 0.09, according to Wilks Lambda.

Follow-up analyses revealed that the unbiased hit rates of younger participants were significantly above chance levels for all three smile types [positive-affect smiles: *F*_(1, 47)_ = 46.56, *p* = 0.000, partial η^2^ = 0.50; negative-affect smiles: *F*_(1, 47)_ = 25.40, *p* = 0.000, partial η^2^ = 0.35; neutral smiles: *F*_(1, 47)_ = 75.05, *p* = 0.000, partial η^2^ = 0.62]. Older adults' unbiased hit rates, however, were only significantly better than chance in correctly identifying neutral smiles [*F*_(1, 47)_ = 14.30, *p* = 0.000, partial η^2^ = 0.22], but did not differ significantly from chance levels for positive-affect and negative-affect smiles (all *p* > 0.05).

### Summary of central findings in study 1

Study 1 revealed that younger and older participants differed from each other in their tendencies to endorse the available response options for categorizing smile expressions, irrespective of whether the endorsed response was correct. Younger participants chose the category “positive-affect smile” significantly more frequently, and the category “neutral smile” significantly less frequently than older participants did. Younger and older participants did not differ from each other in the frequency of categorizing smile expressions as “negative-affect smile.”

In addition, younger participants had higher unbiased hit rates than older adults for categorizing younger targets' smile expressions as positive-affect, negative-affect, or neutral smiles. In fact, older participants' unbiased hit rates for positive-affect and negative-affect smiles were not significantly different from performance levels that were to be expected by chance, given the participants' use of the response options. Younger participants' unbiased hit rates, in contrast, were significantly above chance-level performance for all three types of smiles. Control analyses showed that the age difference in unbiased hit rates was robust to simultaneously controlling for age-related differences in years of education, processing speed, vocabulary knowledge, and vision.

## Study 2

The purpose of Study 2 was to replicate findings from Study 1 and to investigate whether they were moderated by the age of the smiling persons. To fulfill this purpose, the smile task in Study 2 involved distinguishing between positive-affect and neutral smile expressions shown by younger and older targets. Negative-affect smiles were available only from younger targets and were thus not included in Study 2.

### Sample

The sample consisted of *N* = 97 participants living in or around Berlin, Germany. Participants were recruited from a participant database of the Max Planck Institute for Human Development, Berlin. Requirements for participation were the same as in Study 1. In addition, we ensured that none of the participants had taken part in Study 1. Younger participants were between 20.2 and 30.9 years of age (*n* = 48, *M* = 25.67, *SD* = 2.72); older participants were between 70.0 and 78.8 years of age (*n* = 49, *M* = 73.55, *SD* = 2.53). Both participant age groups were approximately stratified by gender (50 and 51% female in the younger and older age groups, respectively) and education (52.1 and 38.8% with German university-entrance qualification in the younger and older age groups, respectively). Informed consent was obtained from all participants, and the ethics committee of the Max Planck Institute for Human Development had approved of the study.

### Measures

#### Smiles task

With two exceptions, the smiles task followed the same logic as in Study 1. The first difference involves the smile stimuli presented. In Study 2, participants watched 64 video recordings of positive-affect smiles shown by younger targets (*n* = 16) and older targets (*n* = 16), and of neutral smiles shown by younger targets (*n* = 16) and older targets (*n* = 16). The second difference involved the number of response options. This time, participants completed the sentence stem “The person smiled in a situation …” by choosing one of two response options: (a) “in which he or she experienced a pleasant feeling (e.g., amusement),” or (b) “in which he or she posed a smile without feeling anything.”

#### Control variables

Information on *years of education* (self report), *perceptual-motor speed* (Digit Symbol Substitution Test), *vocabulary knowledge* (MWT-A), and *visual contrast sensitivity* (FrACT) were obtained as covariates. The measures for these variables were the same as in Study 1. In Table 5 the younger and older subsample are compared on these control variables. As in Study 1, the overall pattern of age differences is consistent with what would be expected based on the developmental literature. Younger participants reported fewer years of professional training, obtained higher scores in the perceptual-speed task, lower scores in the vocabulary task, and had better visual contrast sensitivity than older participants did. Younger and older participants did not differ significantly with regard to years of school education.

**Table 5:** **Descriptives of control variables in Study 2**.

**Construct**	**Younger participants (perceiver)**		**Older participants (perceiver)**				
	***M***	***SD***	***M***	***SD***	***F***	***df***	***p***
Years of school education	11.81	1.55	11.04	2.29	3.674	1, 94	0.058
Years of professional training	3.34	2.02	8.16	9.78	10.431	1, 86	0.002
Perceptual-motor speed (Digit-Symbol)	58.94	10.67	41.80	10.19	65.467	1, 95	0.000
Vocabulary knowledge (MWT-A)	29.65	2.77	31.84	2.68	15.669	1, 95	0.000
Contrast sensitivity (Michelson contrast)^a^	0.67	0.31	2.82	3.13	22.444	1, 95	0.000

*Multivariate age group effect according to Wilks Lambda: F_(4, 81)_ = 25.79, p = 0.000, partial η^2^ = 0.61*.

a *Smaller values indicate better contrast sensitivity*.

### Results

We present the results of Study 2 following the same logic as in Study 1: First we report analyses on age-related differences in participants' response tendencies when evaluating the emotional experience accompanying smile expressions, irrespective of whether these responses were correct. Then we report age differences in the unbiased hit rates for identifying emotional experiences accompanying smiles, and analyze whether the observed unbiased hit rates were significantly different from chance-level performance.

#### Response tendencies

**Figure 3:**
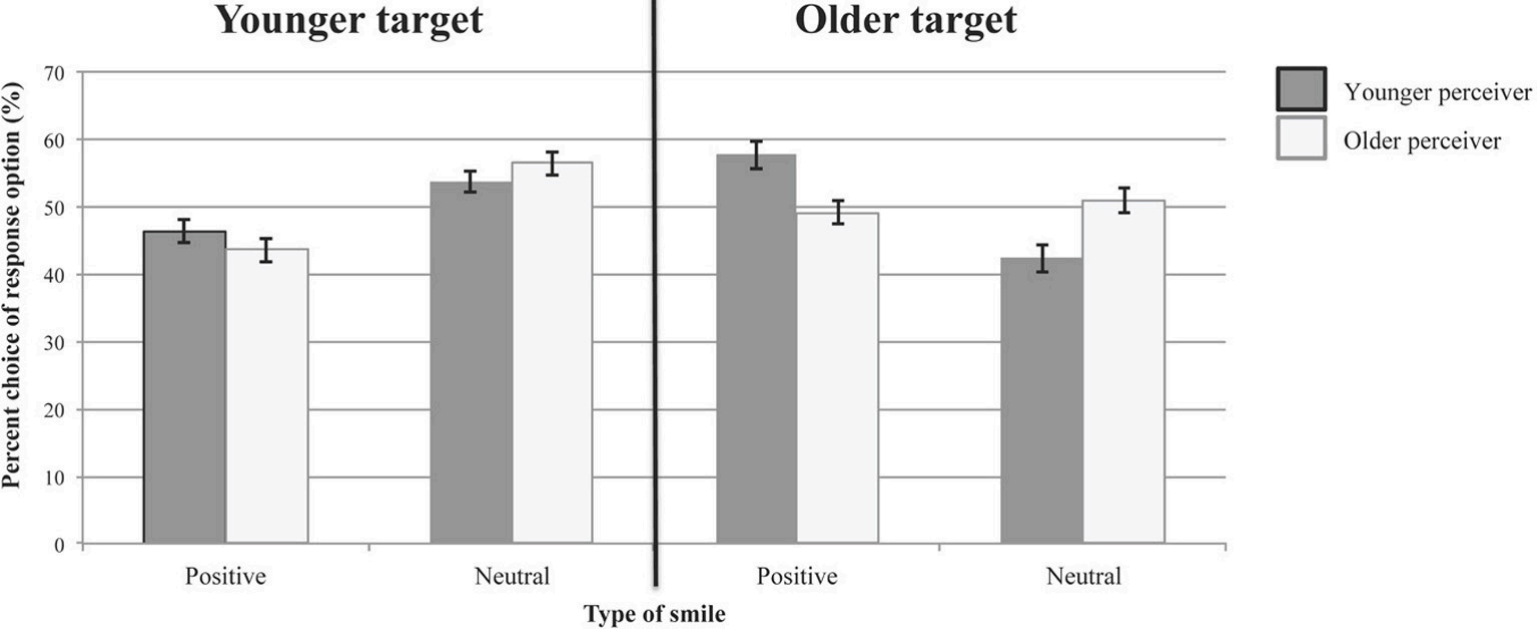
**Use of response options in Study 2 (irrespective of whether response choice was correct)**. Error bars represent ±1 standard errors from the mean.

Figure 3 shows the percentages with which younger and older participants chose each response option in evaluating the emotional experience accompanying the smile expressions, irrespective of whether or not the chosen response was correct. The results of a multivariate analysis of variance on the percentage for choosing the response option “positive-affect smile,” with age group of participants (younger and older) as between-person factor, and age group of target (younger and older smiling persons) as within-person factor are summarized in Table 6. (The pattern of findings for the other response option “neutral smile” is complementary.) Both main effects were significant, as was the Age of Target × Age of Participant interaction. Follow-up analyses indicated that both younger and older participants chose the response option “positive-affect smile” more often for smile expressions shown by older as compared to younger targets: *F*_(1, 47)_ = 56.60, *p* = 0.000, partial η^2^ = 0.55 and *F*_(1, 48)_ = 16.69, *p* = 0.000, partial η^2^ = 0.26, respectively. This effect, however, was more pronounced for younger than for older participants (see Figure 3). Younger and older participants did not differ in how often they chose the response option “positive-affect smile” for expressions shown by younger targets, *F*_(1, 47)_ = 1.30, *p* = 0.257, partial η^2^ = 0.01. They differed, however, with regard to their response preferences for older targets, with younger participants choosing the response option “positive-affect smile” more frequently than older participants, *F*_(1)_ = 9.92, *p* = 0.002, partial η^2^ = 0.10. When judging smile expressions from younger target persons, both younger and older participants chose the response option “neutral smile” significantly more often than in 50% of the cases, and thus significantly more frequently than the response option “positive-affect smile,” one-sample *T*_(47)_ = 2.27, *p* = 0.028 and one-sample *T*_(48)_ = 3.64, *p* = 0.001, respectively. When judging smile expressions from older target persons, however, younger participants chose the response option “positive-affect smile” more frequently than in 50% of the cases, and hence significantly more often than the response option “neutral smile,” one-sample *T*_(47)_ = 3.75, *p* = 0.000. Older participants, in contrast, chose both response options about equally often when judging smile expressions of older target persons, one-sample *T*_(48)_ = −0.49, *p* = 0.627. These findings indicate differences between age group of participants in the preferential use of response categories and thus again underscore the importance of using the unbiased hit rate (Wagner, 1993) when analyzing how well participants from different age groups were able to identify emotional experiences accompanying smiles shown by targets of different age groups.

**Table 6:** **Predicting choice of response option “positive-affect smile” (irrespective of whether response choice was correct) in Study 2**.

**Effect**	***F***	***df***	***p***	**Partial eta squared**
Age group of target^a^	69.258	1, 95	0.000	0.422
Age group of target × Age group of perceiver^a^	8.034	1, 95	0.006	0.078
Age group of perceiver	5.733	1	0.019	0.057

a *Multivariate F-test based on Wilks Lambda*.

#### Age differences in unbiased hit rate for identifying emotional expressions accompanying smiles

**Figure 4:**
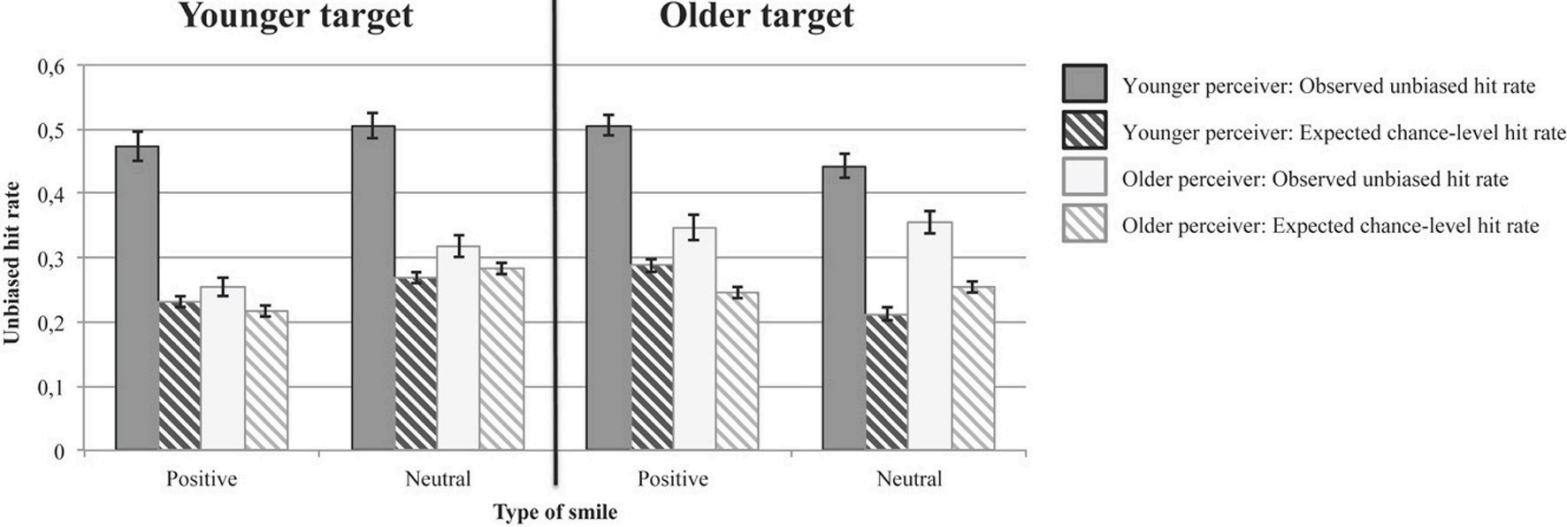
**Observed unbiased hit rates and expected chance-level hit rates in Study 2**. Error bars represent ±1 standard errors from the mean.

Solid bars in Figure 4 represent the average unbiased hit rates in the younger and older participants for correctly identifying positive-affect and neutral smiles shown by younger and older targets. Striped bars in Figure 4 indicate average expected chance levels of performance. In the following, we first analyze age-related differences in unbiased hit rates, and then investigate whether the observed unbiased hit rates were significantly different from chance-level performance.

Table 7 summarizes the results of a multivariate analysis of variance on the unbiased hit rate with age group of participants (younger and older) as between-person factor, and smile type (positive-affect smile and neutral smile) and age group of target (younger and older) as within-person factors. This analysis yielded a significant Age Group of Target × Age Group of Participant × Type of Smile interaction. Consistent with our prediction, follow-up analyses revealed that the Age Group of Target × Age Group of Participant interaction was significant both for positive-affect smiles, *F*_(1, 95)_ = 4.74, *p* = 0.032, partial η^2^ = 0.048, and for neutral smiles, *F*_(1, 95)_ = 12.30, *p* = 0.001, partial η^2^ = 0.12. With regard to positive-affect smiles, older participants were more accurate in identifying positive-affect smiles shown by older as compared to those shown by younger targets, *F*_(1, 48)_ = 21.27, *p* = 0.000, partial η^2^ = 0.31; whereas younger participants' unbiased hit-rates for identifying positive-affect smiles was independent of the age of the smiling person, *F*_(1, 47)_ = 2.77, *p* = 0.103, partial η^2^ = 0.06. For the neutral smiles, older perceivers were again more accurate in identifying neutral smiles posed by older as opposed to by younger targets, *F*_(1, 48)_ = 4.10, *p* = 0.049, partial η^2^ = 0.08; whereas younger perceivers were more accurate at identifying neutral smiles posed by younger than by older targets, *F*_(1, 47)_ = 8.19, *p* = 0.006, partial η^2^ = 0.15.

**Table 7:** **Predicting unbiased hit rates in Study 2**.

**Effect**	***F***	***df***	***p***	**Partial eta squared**
Age group of target^a^	3.295	1, 95	0.073	0.034
Type of smile^a^	0.921	1, 95	0.340	0.010
Age group of perceiver	68.753	1	0.000	0.420
Age group of target × Age group of perceiver^a^	9.024	1, 95	0.003	0.087
Type of smile × Age group of perceiver^a^	5.977	1, 95	0.016	0.059
Age group of target × Type of smile^a^	72.971	1, 95	0.000	0.434
Age group of target × Type of smile × Age group of perceiver^a^	4.810	1, 95	0.031	0.048

a *Multivariate F-test based on Wilks Lambda*.

Compared to older participants' unbiased hit rates, younger participants' unbiased hit rates were generally higher, which is contrary to our hypotheses and to results from previous studies, but consistent with the findings in Study 1. Corresponding to our prediction, however, the size of these age differences in unbiased hit rates was more pronounced for smile expressions shown by younger targets than for smile expressions shown by older targets (age-of-participant effect for positive-affect smiles shown by younger targets: *F*_(1)_ = 66.67, *p* = 0.000, partial η^2^ = 0.41; for positive-affect smiles shown by older targets: *F*_(1)_ = 37.06, *p* = 0.000, partial η^2^ = 0.28; for neutral smiles posed by younger targets: *F*_(1)_ = 49.49, *p* = 0.000, partial η^2^ = 0.34; and for neutral smiles posed by older targets, *F*_(1)_ = 11.17, *p* = 0.001, partial η^2^ = 0.11).

All effects of relevance for our predictions remained robust after controlling for participants' years of education, processing speed, vocabulary knowledge, and visual contrast sensitivity. In other words, the main effect of age group of participants remained significant, *F*_(1)_ = 41.65, *p* = 0.000, partial η^2^ = 0.31, as did the Age Group of Target × Age Group of Participant interaction, *F*_(1, 91)_ = 4.91, *p* = 0.029, partial η^2^ = 0.05 according to Wilks Lambda, and the Type of Smile × Age Group of Participant interaction, *F*_(1, 91)_ = 6.62, *p* = 0.012, partial η^2^ = 0.07 according to Wilks Lambda. The other significant effects shown in Table 7 ceased to reach significance in this control analysis, all *p* > 0.05.

To investigate whether the observed unbiased hit rates differed significantly from chance-level performance, we again followed the procedure proposed by Wagner (1993). As in Study 1, we determined, separately for each participant, the unbiased hit rates that were to be expected by chance given the participant's use of response options. These chance probabilities for correct responses are depicted as striped bars in Figure 4. Observed unbiased hit rates for positive-affect smiles were significantly larger than the respective expected chance-level probabilities. This was the case in both age groups of participants and for positive-affect and neutral smile expressions shown by younger and older targets, respectively [effects of observed vs. chance-level hit rates for positive smiles by younger targets: *F*_(1, 47)_ = 141.71, *p* = 0.000, partial η^2^ = 0.75 for younger and *F*_(1, 48)_ = 7.69, *p* = 0.008, partial η^2^ = 0.14 for older participants, respectively; for positive smiles by older targets: *F*_(1, 47)_ = 200.90, *p* = 0.000, partial η^2^ = 0.81 for younger and *F*_(1, 48)_ = 33.10, *p* = 0.000, partial η^2^ = 0.41 for older participants, respectively; for neutral smiles by younger targets: *F*_(1, 47)_ = 134.09, *p* = 0.000, partial η^2^ = 0.74 for younger and *F*_(1, 48)_ = 7.21, *p* = 0.010, partial η^2^ = 0.13 for older participants, respectively; and for neutral smiles by older targets: *F*_(1, 47)_ = 207.66, *p* = 0.000, partial η^2^ = 0.82 for younger and *F*_(1, 48)_ = 34.27, *p* = 0.000, partial η^2^ = 0.42 for older participants, respectively; all according to Wilks Lambda].

### Summary of central findings in study 2

Younger and older participants in Study 2 did not differ from each other in their response tendencies in evaluating smiles of younger target persons, and chose the response option “neutral smile” more frequently for younger targets' smile expressions than the response option “positive-affect smile.” Furthermore, both younger and older participants ascribed positive-affective experiences more frequently to smile expressions shown by older as compared to younger target persons, but this age-of-target effect was particularly pronounced among younger participants. This was because younger participants in Study 2 chose the response option “positive-affect smile” more frequently and the response option “neutral smiles” less frequently than older participants did when evaluating the emotional nature of smile expressions displayed by older target persons.

Unbiased hit rates for younger and older participants were significantly above chance levels for all smile types. Having to choose between two categories of smile expressions in Study 2 was obviously an easier task than the three-fold categorization required in Study 1. (The similarity of both samples with regard to education, cognitive abilities, and vision that is evident in Tables 2 and 5 renders the possibility unlikely that the difference in task performance is due to differences between the two samples tested.) Despite this difference in task difficulty, Study 2 replicated the better performance of younger participants for correctly identifying all types of smiles. It also indicated, however, that the size of this age difference was moderated by the age of the target person, such that age differences in unbiased hit rates were more pronounced for smile expressions from younger targets and less pronounced for those from older targets. As in Study 1, the age differences in unbiased hit rates were robust to simultaneously controlling for age-related differences in years of education, processing speed, vocabulary knowledge, and vision.

## Discussion

Smile expressions can accompany diverse emotional experiences, such as amusement or anger, but can also occur in the absence of intense emotions. The current study contributes to an emerging line of research on adult age differences in the ability to identify the emotional meaning of other people's smile expressions. We compiled a set of 80 dynamic smile expressions displayed in different contexts by adults of different age groups whose self-reported affective experience matched the intended emotional nature of the situation. Comparisons of the target person's self-reported affective experiences while smiling in the three different contexts (i.e., while being the target of an unfair accusation, while watching amusing material, and while being instructed to pose a smile) demonstrated that we had successfully compiled groups of three emotionally distinct types of smile expressions (i.e., negative-affect, positive-affect, and neutral smiles).

FACS-coding of the Duchenne marker (AU6, Ekman and Friesen, 1978) confirmed prior evidence that contraction of the lateral part of the muscle surrounding the eyes does not reliably distinguish between the different emotional experiences that can accompany smile expressions (for overviews, see Messinger et al., 1999; Krumhuber and Manstead, 2009). Contrary to the assumption in many previous studies that activation of the Duchenne marker is an indicator of positive affect, we found that it was not reliably present in our selection of positive-affect smiles, and not reliably absent in our selection of negative-affect and neutral smiles. On the contrary, substantial proportions of the smile expressions in all three smile categories involved activation of the Duchenne marker. A limitation of our study was that only younger adults conducted the FACS-coding. In future studies it would be desirable to obtain FACS codings from older adult raters as well.

We used this newly developed set of content-valid and dynamic smile stimuli as stimulus material in two studies. In Study 1, we investigated potential differences between younger and older adults in their ability to distinguish positive-affect, negative-affect, and neutral smile expressions shown by younger target persons. In Study 2, we investigated the potential role of the target persons' age by investigating younger and older adults' ability to identify the emotional experiences accompanying positive-affect and neutral smile expressions shown by both younger and older targets.

### Age differences in use of response options (irrespective of whether responses were correct)

In both studies, younger and older participants differed in their tendencies to endorse the available response options, irrespective of whether the endorsed response was correct. It is worth noting that the pattern of response preferences was different from previous findings of an age-related increase in preferential attention to and memory for positive over negative information (for overviews, see Carstensen and Mikels, 2005; Reed and Carstensen, 2012), which are also reflected in the ways how adults from different age groups interpret still pictures of posed expressions of highly intense basic emotions as used in the traditional paradigm (Riediger et al., 2011). The present studies demonstrate that such positivity effects do not generalize to how older adults interpret the emotional meaning of other people's dynamic smile expressions. In fact, the observed pattern for reading smile expressions was in part opposite to what has been found in the domain of attention and memory or the reading of emotional poses in the traditional paradigm: older participants ascribed *less* positive affective experiences to smile expressions than younger participants did. Of interest is also the observation that participants' response tendencies varied depending on the age of the smiling person. Both younger and older participants ascribed positive affective experiences more frequently to smile expressions shown by older target persons than to smile expressions shown by younger adults. The underlying causes remain to be investigated in future studies. They may include characteristics of the smiling persons, such as aging-related differences in skin texture and facial appearance, but also characteristics of the perceiving persons (participants), such as their subjective theories of cohort differences in emotional expressiveness (Otta, 1998). Indeed, social conventions for when smiling is appropriate and expected have changed throughout the 20th century. This is evident, for example, in a greater likelihood for younger as opposed to older cohorts to present themselves smiling in wedding or yearbook photographs (DeSantis and Sierra, 2000). The present results indicated that people were, in fact, more inclined to assume that a smile expression is emotionally neutral when it was displayed by a younger than by an older adult. This effect was particularly pronounced among the younger participants, which could reflect an age difference in subjective theories about the frequency with which older adults show emotionally neutral smile expressions, and possibly arises from differences in the frequency of contact with older individuals.

The observed age differences in response tendencies are interesting not only because they hint at possible age differences in people's subjective theories about the emotional nature of smile expressions. These results also underscore the importance of accounting for the potentially distorting effects of response tendencies on accuracy indices of smile categorizations, because the more often a particular response option is chosen, the higher the unbiased likelihood is to correctly categorize smile stimuli that belong to that smile type.

### Age differences in the accuracy of identifying emotional experiences from facial expressions

We determined participants' accuracy of smile categorizations as unbiased hit rates according to Wagner (1993). This indicator removes the potentially biasing effects of response tendencies by determining the joint probabilities that a given participant had correctly identified the smile type and that she had correctly used the respective response category. Contrary to our expectations and to evidence from previous studies on age differences in identifying the emotional meaning of smile expressions (McLellan, 2008; Murphy et al., 2010; Slessor et al., 2010a), younger participants outperformed older participants in both studies and with regard to all investigated types of smile. Corresponding to our hypotheses, however, older participants in Study 2 were more accurate in identifying the emotional meaning of smile expressions shown by older as compared to younger targets, which could have resulted from their better knowledge, or greater experience with smile expressions shown by older as opposed to younger individuals (e.g., Harrison and Hole, 2009). For younger participants, a corresponding own-age advantage was evident only in identifying neutral smiles; that is, younger participants were better at identifying neutral smile expressions stemming from younger target persons as opposed to older target persons. A limitation of the present research, which future studies should remedy, is that we were not able to investigate whether similar own-age advantages would also be evident for negative-affect smiles.

The observed own-age advantage for older participants' accuracy in identifying the emotional meaning of neutral and positive-affect smile expressions supports the argument that age differences in recognizing emotion from facial expressions are likely to be over-estimated in studies that only consider facial expressions from younger or middle-aged, but not older target persons. The unbiased hit rates of older participants were smaller than those of younger participants, however, even for smile expressions from the older targets. Overall, the present research thus supports the view that the ability to decipher emotional meaning from facial expressions alone, presented without the accompanying context, is not as good in older than for in younger adults. Control analyses in both studies showed that the age differences in unbiased hit rates were robust to simultaneously controlling for age-related differences in years of education, processing speed, vocabulary knowledge, and vision, showing that differences in these control variables did not account for the observed age differences in reading smiles. It thus remains an open question for future research to identify the mechanisms that underlie these effects. Future studies should examine, for example, the potential respective roles of age-related differences in subjective conceptions of when and by whom smile expressions are displayed, or in the ability to integrate information from various (e.g., structural and temporal) dimensions of smile expressions (Slessor et al., 2010b).

The results from the present studies are strikingly different from those of earlier studies that used smile stimuli. It seems likely that this is due to diverging strategies of compiling smile expressions. In contrast to the previous investigations, our collection of smile stimuli was both dynamic and content-valid in the sense that we selected smile expressions for different smile types based on the convergence between the affective nature of the situation in which the smile was shown (i.e., while being the target of an unfair accusation, watching amusing stimuli, or being asked to pose a smile) and the target persons' self-reported affective experiences in that situation. The earlier studies, in contrast, used the Duchenne marker either as the sole criterion for the categorization of smile expressions (Murphy et al., 2010), or as one criterion among several (McLellan, 2008; Slessor et al., 2010a). In replication of evidence from other studies, all categories of our content-valid selection of smile expressions included a substantial proportion of stimuli that involved the Duchenne marker, suggesting that the Duchenne marker is not a reliable criterion for distinguishing between different types of smiles. Another possible explanation for the disparity of findings could relate to the different cultural contexts (i.e., in Germany, the US, and the UK) in which the respective investigations had been conducted. An interesting task for future investigations remains to explore whether cultural differences in smile expressions, or in smile recognition, may have contributed to the observed differences in findings. Another open question for further studies is the identification of contexts in which the observed age differences in recognizing affective experiences that accompany smile expressions might be reduced (or perhaps even reversed). One might speculate, for example, that familiarity with the smiling person might play a moderating role in this regard.

Future research should also aim at overcoming some methodological limitations of the present research. Among the most notable of these is the present cross-sectional design. Future research should explore the extent to which the observed cross-sectional differences between age groups reflect cohort differences as opposed to aging-related changes within persons over time. Another important task for future research is closing the gap to real-life emotion-recognition demands: even though the smiles paradigm (in contrast to the traditional paradigm) employs expressions that participants frequently encounter in diverse emotional contexts of their daily lives, it is nevertheless still quite different from real-life emotion-recognition demands. One obvious difference is that real-life smile expressions occur within the context of a particular situation and that perceivers might have accumulated knowledge about the smiling person. Investigating potential age-related differences in the ability to decipher smile expressions when such contextual information is available remains an open task for future research. Another realm for future investigation would be to further increase the breadth of smile types under investigation, and to also consider the role of arousal. In the present study, we focused on smile expressions that accompanied emotional states that were unambiguous with regard to their valence (negative, positive, neutral); however, different elicitation methods were employed to obtain each of the three smile types, making it possible that the smile stimuli might differ in other factors than valence as well. Furthermore, the positive-affect and negative-affect smiles were recorded in potentially activating emotional contexts (i.e., anger and amusement). This leaves an open question as to whether the observed age differences would generalize to smile expressions accompanying other emotional states, for example, mixed emotional states (e.g., feeling joyful and sad at the same time) or low-arousal states (e.g., sadness or contentment).

### Summary and conclusion

Two studies provided converging evidence that younger adult participants were better able to identify the emotional experiences accompanying different types of smile expressions than older participants were. Results further showed that these age differences were attenuated for smile expressions displayed by older target persons. Older adults were better able to identify the emotional meaning of smile expressions shown by older as compared to younger target persons. We conclude that dynamic and content-valid smile expressions provide a promising venue for studying age-differences in emotion recognition, and that it is important to vary the age of the expressing persons to further the understanding of adult age differences in the ability to recognize the emotional meaning of facial expressions.

### Conflict of interest statement

The authors declare that the research was conducted in the absence of any commercial or financial relationships that could be construed as a potential conflict of interest.

## Footnotes

^1^This was due to differential patterns within age groups for the relative accuracies in identifying the different types of smiles. Younger adults' unbiased hit rates for identifying negative-affect smiles were significantly lower than those for identifying positive-affect [*F*_(1)_ = 14.63, *p* = 0.000, partial η^2^ = 0.24] and neutral smiles [*F*_(1)_ = 27.84, *p* = 0.000, partial η^2^ = 0.37], which did not differ significantly from each other (*p* > 0.05). Among older adults, the unbiased hit rate for negative-affect smiles did not differ significantly from that for positive-affect smiles (*p* > 0.05), and unbiased hit rates for both negative-affect and positive-affect smiles were significantly lower than the unbiased hit rate for neutral smiles [positive smile: *F*_(1)_ = 24.81, *p* = 0.000, partial η^2^ = 0.33]; negative smiles [*F*_(1)_ = 18.04, *p* = 0.000, partial η^2^ = 0.26]. Note, however, that this age group × smile type interaction did not remain significant in our control analysis.
